# Circadian Integration of Glutamatergic Signals by Little SAAS in Novel Suprachiasmatic Circuits

**DOI:** 10.1371/journal.pone.0012612

**Published:** 2010-09-07

**Authors:** Norman Atkins, Jennifer W. Mitchell, Elena V. Romanova, Daniel J. Morgan, Tara P. Cominski, Jennifer L. Ecker, John E. Pintar, Jonathan V. Sweedler, Martha U. Gillette

**Affiliations:** 1 Neuroscience Program, University of Illinois, Urbana, Illinois, United States of America; 2 Department of Cell and Developmental Biology, University of Illinois, Urbana, Illinois, United States of America; 3 Beckman Institute for Advanced Science and Technology, University of Illinois, Urbana, Illinois, United States of America; 4 Department of Cell Biology and Neuroscience, University of Medicine and Dentistry of New Jersey, Newark, New Jersey, United States of America; 5 Department of Biology, The Johns Hopkins University, Baltimore, Maryland, United States of America; 6 Department of Chemistry, University of Illinois, Urbana, Illinois, United States of America; Vanderbilt University, United States of America

## Abstract

**Background:**

Neuropeptides are critical integrative elements within the central circadian clock in the suprachiasmatic nucleus (SCN), where they mediate both cell-to-cell synchronization and phase adjustments that cause light entrainment. Forward peptidomics identified little SAAS, derived from the proSAAS prohormone, among novel SCN peptides, but its role in the SCN is poorly understood.

**Methodology/Principal Findings:**

Little SAAS localization and co-expression with established SCN neuropeptides were evaluated by immunohistochemistry using highly specific antisera and stereological analysis. Functional context was assessed relative to c-FOS induction in light-stimulated animals and on neuronal circadian rhythms in glutamate-stimulated brain slices. We found that little SAAS-expressing neurons comprise the third most abundant neuropeptidergic class (16.4%) with unusual functional circuit contexts. Little SAAS is localized within the densely retinorecipient central SCN of both rat and mouse, but not the retinohypothalamic tract (RHT). Some little SAAS colocalizes with vasoactive intestinal polypeptide (VIP) or gastrin-releasing peptide (GRP), known mediators of light signals, but not arginine vasopressin (AVP). Nearly 50% of little SAAS neurons express c-FOS in response to light exposure in early night. Blockade of signals that relay light information, via NMDA receptors or VIP- and GRP-cognate receptors, has no effect on phase delays of circadian rhythms induced by little SAAS.

**Conclusions/Significance:**

Little SAAS relays signals downstream of light/glutamatergic signaling from eye to SCN, and independent of VIP and GRP action. These findings suggest that little SAAS forms a third SCN neuropeptidergic system, processing light information and activating phase-shifts within novel circuits of the central circadian clock.

## Introduction

The hypothalamic suprachiasmatic nucleus (SCN) orchestrates daily rhythms in brain and body functions and aligns them with environmental day and night, processes fundamental to health and longevity [1]. Functions coordinated by this master circadian clock are diverse: behavioral rhythms of activity and rest, peak performance in memory acquisition, release of pituitary hormones into the portal system, oscillations in autonomic functions, time of stem cell migration from the bone marrow into the blood stream, and even disease vulnerabilities. Circadian timekeeping is a cellular phenomenon, so the SCN must integrate its component cellular clocks into a tissue-level clock and transmit coordinate time-of-day information [2]. Because we live in an ever-changing world, the SCN also must integrate ambient timing cues and, if necessary, adjust its time-base to re-align brain and body processes. These requirements impose exceptional modulatory demands at the input, intra-SCN and output levels on the neurons and circuits that comprise the master clock. Thus, neuropeptides are predicted to participate in SCN functions to an unusual degree [3].

The internal structure of the SCN is complex [4]. Phenotypes and functional roles of the ∼10,000 SCN cells are incompletely understood, although evidence indicates they are related to neuropeptide expression [5]. Generalizations distilled from localization patterns of peptides first described have been applied to conceptually organize the SCN into a dichotomous structure. Nevertheless, assessments based on differential and dynamic peptide localizations, distribution and density of afferent projections, three-dimensional topography and rhythmic expressions of clock genes and neuronal activities indicate that the SCN may be significantly more complex [3], [6], [7], [8], [9], [10], [11], [12]. The general patterning of these features is conserved across mammalian species, suggesting that such complexity is fundamental to SCN functional organization [6], [13], [14], [15], [16].

Whereas only VIP and GRP have established roles in synchronizing cellular oscillators and integrating/relaying light information within the SCN, recent mass-spectrometric analyses of the SCN peptidome report that peptide expression and release are much more extensive and diverse than recognized previously [3], [7]. The SCN was found to express 102 neuropeptides identified positively by mass spectrometry, but the functions of most are unknown [11], [15], [17]. Why does the SCN express and release such an abundance of neuropeptides? Elucidating the spatiotemporal and functional contexts of newly identified peptides has emerged as the challenge to fully understanding SCN physiology.

Little SAAS emerged through our high-throughput peptidomics studies as a potentially prominent SCN neuropeptide. Derived from the proSAAS prohormone (pcsk1n; UniProt Assession # Q9QXU9) [18], which has been proposed to function as a prohormone-processing enzyme, little SAAS was among the more abundant neuropeptides detected in SCN releasate [3], [7]. Its levels change in the supraoptic nucleus in response to dehydration, but function is not established [19]. An initial peptidomics study reported that little SAAS is secreted spontaneously with circadian rhythmicity from an SCN brain slice, where stimulating optic nerve (ON) evokes little SAAS release and exogenous little SAAS can alter endogenous rhythms of neuronal single unit activity (SUA) [7]. Nevertheless, these observations do not establish little SAAS as an endogenous SCN neuropeptide or a contributor to circadian function in this complex tissue. The aim of our study is to evaluate little SAAS localization, functional context and integrative physiology with respect to known mediators of cell-to-cell signaling in the SCN in order to better understand the relative importance of this new SCN peptide discovered by cutting-edge analytical chemistry.

## Methods

### Ethics statement

All experimental procedures were conducted at the University of Illinois at Urbana-Champaign under protocols approved in advance by the Institutional Animal Care and Use Committee (IACUC) under Animal Welfare Assurance number A3118-01. All care and experiments were conducted in full compliance with principals and procedures outlines in the National Institutes of Health Guide for the Care and Use of Laboratory Animals.

### Animals and circadian time

Seven- to thirteen-week old Long Evans/BluGill rats were used in this study. This inbred strain has been analyzed via dense genomic scan (10 cM inter-marker interval), which confirmed only one allele at each locus examined [20]. Animals were housed in 12∶12 h light/dark conditions with constant temperature and humidity, food and water *ad libitum*. Circadian time (CT) designates intrinsic time-of-day regulated by the near-24-h endogenous oscillation of the SCN. In the SCN brain slice, CT is an indicator of subjective day and night for determining circadian time-points for exogenous treatments and of endogenous clock state [21].

All mice used had a C57BL6 genetic background, including controls, animals heterozygous for melanopsin-Cre (*Opn4-Cre*) and Z/EG [22], and proSAAS +/y and −/y. The ProSAAS null was created by excision of proprotein convertase subtilisin/kexin type 1 inhibitor (*pcsk1n*) exon 1 and homologous recombination [23]. ProSAAS mouse brain tissue was prepared at the University of Medicine and Dentistry of New Jersey and shipped to the University of Illinois at Urbana-Champaign for processing.

### Preparation of brain slices

Experiments utilized 500-µm coronal brain slices containing the mid-SCN, prepared during daytime ≥2 h before the onset of the dark phase, as preparation at night can alter clock phase [24], [25]. Hypothalamic tissue was isolated from the brain, and coronal slices were cut at 500 µm by mechanical chopper. Slices containing the SCN were maintained in a brain slice chamber, kept at constant temperature and gas conditions, perifused with glucose-/bicarbonate-/gentamicin-supplemented Earle's balanced salt solution (EBSS, Hyclone) at 37°C and saturated with 95% O_2_∶5% CO_2_.

### Brain slice treatments

Treatment of coronal SCN brain slices was performed with perifusion pump stopped and EBSS level lowered slightly to expose the tissue surface. Little SAAS, vasoactive intestinal polypeptide (VIP), gastrin-releasing peptide (GRP), and [4Cl-D-Phe^6^, Leu^17^] VIP were purchased from Phoenix Pharmaceuticals. PD176252 was purchased from Tocris Bioscience. Peptides, including little SAAS (various concentrations) and/or VIP/GRP cocktail (100 nM each), were applied via 1-µL drop directly onto each bilateral SCN visible at the brain slice surface. After 5 min, the surface was rinsed with EBSS, and perifusion was resumed. Antagonists, including little SAAS antiserum #2768 (undiluted) or [4Cl-D-Phe^6^, Leu^17^] VIP/PD176252 antagonist cocktail (50 mM each in 0.05% DMSO), were applied like agonists, 10 min prior to EBSS rinse (control) or subsequent peptide treatment. Effective concentrations for µL treatments are estimated to be diluted 10–100× during diffusion into the tissue.

### Recording of single unit activity (SUA)

Extracellular recording of spontaneous SUA of SCN neurons has been described previously [26]. Briefly, the firing rate of an individual neuron is recorded and its mean SUA determined over 4 min; the electrode is repositioned and the process repeated. Two-hour mean SUAs are plotted *versus* circadian time (CT). Running averages are based on 2-h epochs offset by 15-min intervals. Calculation of circadian phase is based upon CT of peak SUA.

### Intracerebroventricular (*i.c.v.*) cannula implantation and colchicine injections

**Figure 1:**
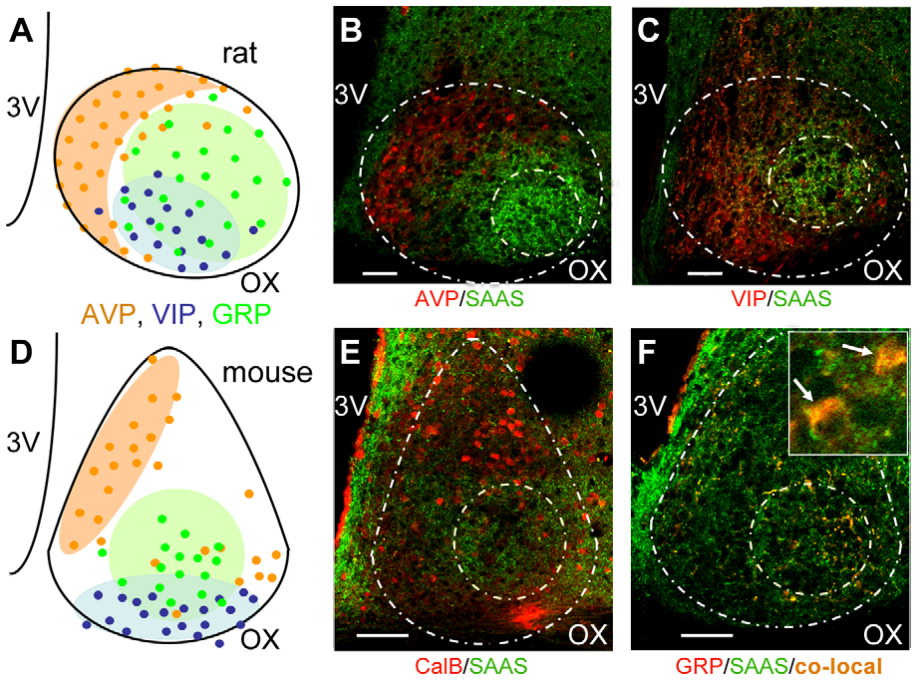
Little SAAS localizes to the central SCN of rat and mouse. Patterns of neuropeptide immunoreactivity in rat and mouse are compared in mid-SCN coronal section. (A, D) Schematic of previously established neuropeptide expression [6], [8], [15] in sub-compartments (shading) and somata (circles): AVP, orange; VIP, blue; GRP, green. (B,C,E,F) Photomicrographs of immunohistochemistry demonstrate that little SAAS localization (green) in rat mid-SCN (B,C) is similar to that observed in mouse SCN (E,F). In both species, little SAAS-positive somata are primarily in the central sub-compartment, although punctuate expression extends more broadly. Little SAAS (green) is shown double-labeled with arginine vasopressin, AVP (B, red), vasoactive intestinal peptide, VIP (C, red), calbindin, CalB (E, red) or gastrin releasing peptide, GRP (F, red). Representative higher magnification (inset, F) of little SAAS (green) and GRP (red) shows significant somatic co-localization (yellow). Each SCN is delineated by an outer dashed line in the shape of an oval (rat) or teardrop (mouse). Localization of strong immunoreactivity for little SAAS is marked by an inner circle, which corresponds to previous descriptions of the central SCN. Images are single optical sections (B,F, z = 0.11µm; F inset, z = 0.09 µm). OX, optic chiasm; SCN, suprachiasmatic nucleus; 3V, third ventricle. Scale bar = 50 µm.

**Figure 2:**
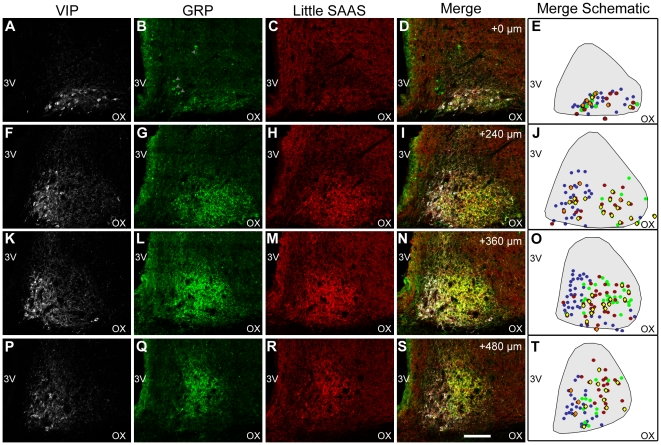
Little SAAS expression spans across VIP and GRP cytoarchitectonic regions of rat SCN. Confocal tile-scan images of coronal tissue sections from rostal-to-caudal quadrants (vertical panels) reveal that little SAAS localizes to VIP- and GRP-expressing sub-compartments in all quadrants. SCN are triple immunostained for VIP (white; A,F,K,P), GRP (green; B,G,L,Q), and little SAAS (red; C,H,M,R). Merge of images in the three channels across each row (D,I,N,S) reveals co-localizations: VIP/little SAAS (pink), VIP/GRP (light green), and GRP/little SAAS (yellow). Enlargements of these merged images appear in supplementary material (Figs. S2, S3, S4, S5). Schematic representations of each merge image (E, J, O, T) mark the location of each stained soma in each optical slice: VIP (blue circles), GRP (green circles), little SAAS (red circles), VIP/little SAAS co-expressing (pink diamonds), and GRP/little SAAS co-expressing (yellow diamonds). Gray arrowheads in B indicate non-specific staining of particulate introduced during tissue mounting. Whereas VIP localizes primarily to the more ventromedial region, GRP occupies the more central SCN, as reported previously. Some overlap with VIP exists along the medial edge of VIP localization, whereas laterally there is considerable overlap with GRP. Non-somatic immunostaining marks peptide localized in cellular processes and puncta. Coronal sections (40 µm) are from colchicine-treated rat. Images in each row are the same optical plane (z = 0.11 µm). Approximate image coordinates provided in I, N, and S are relative to the rostral-most image (D, designated as +0 µm). 3V, third ventricle; OX, optic chiasm. Scale bar (in S) = 100 µm.

Because colchicine has been reported to improve the ability to visualize and count GRP-positive perikarya, which are obscured by a dense fiber plexus [9], we injected colchicine *i.c.v.* in rats when distributions of VIP, GRP and little SAAS would be compared directly (not Fig. 1; Fig. 2 and Table 1, only). Male rats (250–300 g) were administered buprenorphine (0.03 mg/kg; Reckitt Benckiser Healthcare) pre-operatively 1 h prior to *i.p.* injection of ketamine/medetomidine (75 mg/kg and 0.5 mg/kg, Fort Dodge Animal Health and Pfizer Animal Health, respectively). A guide cannula was implanted at stereotaxic coordinates targeting the lateral ventricle (from bregma: A–P −0.9mm, M–L −1.40 mm, D–V −3.5 mm). Animals were revived with atipamezole (1 mg/kg; Pfizer Animal Health) and allowed to recover for at least 10 days under careful observation. Colchicine (5 µL of 10 µg/µL in 0.9% saline; SigmaAldrich) was injected *i.c.v.* into anesthetized, cannulated rats over the course of 5 min. At 36 h post-treatment, animals were deeply anesthetized and brains were prepared for analysis.

**Table 1:** Quantitation of little SAAS-, VIP- and GRP-expressing neurons per rat SCN.

	Number of Neurons per SCN	Estimated Percentage per SCN
Little SAAS	1636±247	16.36%
Vasoactive Intestinal Polypeptide (VIP)	2226±373	22.26%
Gastrin-Releasing Peptide (GRP)	1062±135	10.62%
Little SAAS/VIP	232±65	2.32%
Little SAAS/GRP	842±81	8.42%

Numbers of peptide-expressing neurons are estimated based on comparison of stereological counts to the total number of SCN neurons in colchicine-treated rats. Estimated percentage values are based on an average number of 10,000 neurons per single rat SCN [4], [8]. Little SAAS is co-expressed with VIP or GRP, respectively, in a subset of neurons. However, co-expressing neurons do not account for the total number of little SAAS-positive neurons observed.

### Immunohistochemistry

At 4 h post-lights on, subjects were anesthetized with Euthasol (100 mg/kg, Virbac) and perfused intracardially with 0.9% NaCl followed by 300 ml of 4% paraformaldehyde in 0.1 M PBS. Brains were removed, placed overnight in 4% paraformaldehyde, and then transferred to PBS for 24 h. Coronal sections (40-µm thick) were prepared using a Vibratome® (Leica Microsystems) and rinsed 3×10 min in PBS.

Immunohistochemical examination of little SAAS was conducted using rabbit anti-little SAAS antisera (#2766 or #2768) [27]. For little SAAS immunohistochemistry, sections from untreated rats were incubated with rabbit anti-little SAAS antiserum, 1∶5000 for 48 h at 4°C, and Alexa-Fluor® 568 goat anti-rabbit IgG (Molecular Probes), 1∶1000 for 2 h at room temperature (RT). Normal goat serum (Vector Labs) was used for blocking non-specific staining (5%) and during antibody incubation (2%). For c-FOS/little SAAS immunohistochemistry, sections collected from brains of rats following exposure to light (400 lux, CT 14 for 1 h) were incubated at 4°C in (1) normal bovine serum (5% for 2 h, Jackson Immunoresearch), (2) goat anti-c-FOS (1∶200 for 24 h, Santa Cruz Biotechnology); (3) Biotin-SP-conjugated bovine anti-goat IgG (1∶400 for 1 h, Jackson Immunoresearch), and (4) Fluorescein, DTAF-conjugated streptavidin (6 µg/ml for 1 h, Jackson Immunoresearch). Following these steps, the tissue was processed according to the little SAAS immunihistochemistry protocol described above. PBS (3×10 min) washes at RT were performed between all steps, and PBS with 0.3% Triton X-100 (PBS-T) was used for all serum and antibody dilutions.

Immunohistochemistry involving GRP and/or VIP was performed on colchicine-treated rat brain tissues. For GRP/little SAAS double immunohistochemistry, sections first were blocked for 2 h at RT in normal donkey serum/PBS-T (5%, Vector Labs). Next, sections were incubated sequentially as follows: (1) goat anti-GRP polyclonal antibody (1∶50 for 48 h at 4°C; Santa Cruz Biotechnology), (2) 3×10 min in PBS; (3) Alexa-Fluor® 488 donkey anti-goat IgG (1∶1000 for 2 h at RT, Molecular Probes), (4) 6×10 min PBS; (5) normal goat serum/PBS-T (5%, Vector Labs), (6) rabbit anti-little SAAS antiserum #2766 (1∶5000 for 48 h at 4°C) [27], and (7) Alexa-Fluor® 568 goat anti-rabbit IgG (1∶1000 for 2 h at RT, Molecular Probes). VIP/little SAAS double immunohistochemistry included sequential incubations with (1) normal goat serum/PBS-T (5%, 2 h at RT); (2) guinea pig anti-VIP polyclonal antibody (1∶8000 for 48 h at 4°C, Peninsula Laboratories), (3) 3×10 min in PBS; (4) Alexa-Fluor 488 goat anti-guinea pig IgG (1∶1000 for 2 h at RT, Molecular Probes), (5) 3×10 min PBS; (6) rabbit anti-little SAAS antiserum (1∶5000 for 48 h ); and (7) Alexa-Fluor® 568 goat anti-rabbit IgG (1∶1000 for 2 h at RT, Molecular Probes). Triple immunohistochemistry for GRP, VIP, and little SAAS was performed first with GRP immunohistochemistry, followed by 6×10 min PBS, 5% normal goat serum block for 2 h at RT, and steps (2) through (7), as outlined for VIP/little SAAS double immunohistochemistry above.

Mouse tissue for retinal immunohistochemistry was collected at zeitgeber time 3 (ZT 3, 3 h following light onset). Melanopsin-green fluorescent protein (GFP) transgenic mice were sacrificed by cervical dislocation, and eyes were immediately excised and placed into 4% paraformaldehyde overnight, followed by 24-h incubation in 30% sucrose. Cryo-protected eye tissues were frozen in cryo-molds in O.C.T. Compound (Tissue-Tek; Sakura Finetek). Sections (35 µm) were cut by cryostat and dried for several hours. Sections were post-fixed for 20 min in 4% paraformaldehyde, blocked for 1 h in phosphate buffer containing 0.3% Triton X-100, 2.5% heat-inactivated goat serum (Invitrogen) and 2.5% heat-inactivated donkey serum (Millipore), and then stained for two days in rabbit anti-little SAAS antiserum (1∶4000), and chicken anti-green fluorescent protein polyclonal antibody (1∶1000 at 4°C, Abcam). Incubation with secondary antibodies Alexa-Fluor® 546 goat anti-rabbit IgG (1∶600, Invitrogen) and FITC-conjugated donkey anti-chicken IgY (1∶600, Millipore) was performed for 2 h at RT in the dark. After PBS washes, tissue was cover-slipped in Vectashield fluorescence mounting medium (Vector Laboratories).

For diaminobenzidine (DAB) staining, rat and ProSAAS +/y (WT) and −/y (null) mouse brains were sectioned coronally (40 µm), rinsed 3×10 min in PBS, incubated in 0.3% hydrogen peroxide for 30 min, and rinsed again 3×10 min in PBS at RT. Non-specific staining was blocked using 5% normal goat serum in PBS-T for 60 min at RT. Sections were incubated in rabbit anti-little SAAS serum (1∶32,000 for 48 h) and biotinylated goat anti-rabbit IgG (1∶200; Vector Laboratories) for 60 min, both at 4°C with 3×10 min PBS rinses following primary and secondary antibody incubation. After incubation in the Vector Elite ABC streptavidin horseradish peroxidase complex (1∶28, 120 min at 4°C, Vector Laboratories) and 3×10 min PBS washes, sections were rinsed and developed using DAB, as described previously [28]. After final immunohistochemistry steps, all fluorescence-stained tissue sections were washed 3×10 min in PBS, mounted on SuperFrost Plus microscope slides (ThermoFisher Scientific), dried, and coverslipped with ProLong® Gold antifade reagent (Molecular Probes). Slides from DAB and immunohistochemistry were dehydrated, defatted with Citrisol (ThermoFisher), and coverslipped with Permount (ThermoFisher).

### Imaging

Nissl sections were imaged using a Zeiss Axiolmager A1 light microscope and the image acquisition functions in Stereo Investigator software (MBF Bioscience, Williston, VT). DAB-reacted sections were imaged using a Leica DM 6000B/CTR6000 light microscope under a 3× objective lens by means of the Leica Application Suite software. Fluorescence-immunolabeled images were acquired using a Zeiss LSM-510 confocal microscope. For image acquisition, amplifier gain, offset, respective excitation laser power settings, and scan properties were optimized for each objective lens to neurons displaying the lowest emission levels. Some individual, single-optical slice images were captured as tiled images across two-dimensional space and manually stitched together using Adobe Photoshop CS2 (Adobe Systems, Inc.). No other post-processing of acquired images was conducted. Individual peptide immunolocalizations are represented by red, green, or white; co-localization is represented by pink or yellow. Microscope software-rendered pseudocolor was confirmed as co-localization using ‘Profile’ analysis of captured confocal images (Zeiss Microsystems). Optical slice thickness for confocal images captured with 20×, 40×, and 100× objective lens was 0.45 µm, 0.11, and 0.09 µm, respectively.

### Light pulse for c-FOS induction

Rats were pair-housed in circadian activity monitoring system (CAMS) prior to dark phase onset at ZT 12. At ZT 14, CAMS lights were turned on (400-lux light for 60 min). Animals then were removed from CAMS, euthanized, and perfused according to procedures described above. Brain tissues were collected from light-pulsed rats and compared to brain tissue samples obtained from control animals in darkness at ZT 15.

### SCN cell counting

Total numbers of little SAAS-, GRP-, and VIP-positive neurons in a single, unilateral SCN were determined using a modified version of a stereological method described previously [6]. Serial coronal sections (40 µm) from the hypothalamus of colchicine-treated rats were divided into three groups, referred to as ‘bins’, each containing in a total of six SCN-inclusive sections at intervals 120 µm apart. Sections immunostained for either GRP/little SAAS or VIP/little SAAS were subjected to stereological analysis using a Zeiss AxioImager A1 microscope (Zeiss Microsystems) and Stereo Investigator software (MBF Bioscience).

Total immunopositive cells were counted in six SCN-containing sections from a single bin. Cells of only one SCN per section were counted; counting was performed on the same side of each coronal slice per group, as determined by orientation of the unilateral track mark in the cerebral cortex left by the cannula used for injecting colchicines for *i.c.v.* delivery. Total SCN volume was estimated by measuring the area of a single SCN for each section analyzed and multiplying the area by the thickness of the section. The sum of the volume of all sections counted was multiplied by the total number of groups to obtain the total SCN volume.

We observed that little SAAS immunoreactivity is generally high in the peri-SCN hypothalamus. Little SAAS intensity decreases markedly near the border of the SCN (Fig. S1). SCN volume, determined by circumscribing the perimeter formed by the marked decrease in little SAAS staining at the juxtaposition with the surrounding extra-SCN hypothalamus, was not significantly different from SCN volume calculated using Nissl staining. Thus, this demarcation was used for subsequent determination of SCN volume.

For each sample, the total cell count estimate was calculated as:
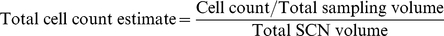
To examine the relationship of little SAAS localization to light-induced c-FOS-like immunoreactivity, coronal brain slices from light pulsed rats were double immunostained, as described above. Immunostained samples were examined using cell counting methods modified from previous studies [29]. For each subject, bilateral SCN from two mid-SCN coronal sections were analyzed using a Zeiss AxioImager A1 microscope (Zeiss Microsystems) and Stereo Investigator software (MBF Bioscience). Little SAAS, c-FOS, and little SAAS/c-FOS double-positive neurons were marked and tallied throughout the entire thickness of respective SCN. Only little SAAS-positive neurons with discernable immunohistochemistry-sparse nuclei were counted, and c-FOS-positive nuclei with discernable nucleoli were counted. The total counts for the four respective SCN from each animal were averaged, and a group average was calculated.

### Mass spectrometry analysis of peptides in rat optic nerve

Optic nerves, from retina to optic chiasm, were dissected from rats immediately after decapitation and processed for peptide extraction as described previously [30]. Briefly, isolated optic nerve samples were placed individually in 20 µL of dihydroxybenzoic acid solution (DHB: 20 mg/mL in water) for preservation and peptide extraction during 48-h incubation at 14°C. Samples for MS were prepared by mixing 0.7 µL of DHB extract 1∶1 with freshly prepared DHB matrix (50 mg/mL, 50% acetone) directly on the MALDI sample plate and analyzed using a Bruker ultraFlex II mass spectrometer (Bruker Daltonik) in positive reflectron mode. Fragmentation spectra of selected peptides were produced from the same samples in tandem MS mode (TOF/TOF), and amino acid sequences were deduced using Bruker Biotools software (Bruker Daltonik) with rapid *de novo* function. Obtained sequence tags were searched against the MSDB and NCBI protein databases via Mascot (Matrix Science, Boston, MA; http://www.matrixscience.com) with 0.5 Da mass tolerance for both parent and fragment ions. Matched peptides were manually inspected for sequence fit.

### Statistical analysis

Analysis of data containing two groups was conducted using Student's *t* test. One-way ANOVA was used for comparison of more than two groups with Bonferroni or Tukey-Kramer post-test. Statistical significance was ascribed at *p*≤0.05.

## Results

### Little SAAS localizes to the central SCN of rat and mouse

We evaluated brain tissues from rat and mouse by fluorescence immunohistochemistry for known peptide markers while staining for little SAAS immunoreactivity. Specificity of little SAAS antisera was demonstrated by loss of immunoreactivity in tissues of proSAAS-deficient transgenic mice (Fig. S1), as well as in rat after pre-incubation of antisera with little SAAS peptide (data not shown). Little SAAS immunoreactivity is evident in the SCN of both rat and mouse (Fig. 1A–F). The shape of the SCN and orientation of core and central sub-compartments differ slightly between these species (Fig. 1A, D). Established geometries and peptidergic markers of sub-compartments were compared to little SAAS localization. At mid-SCN, in both rat and mouse, little SAAS immunoreactivity appears mediolaterally in a circular pattern (Fig. 1B, C, E, F). In both species, little SAAS is not evident in the region marked by arginine vasopressin (AVP) and, in mouse, also, calbindin. Little SAAS localization is less apparent in regions marked by VIP than in the central area marked by GRP in these mid-SCN sections.

To more thoroughly evaluate localization of little SAAS with respect to VIP and GRP, tile scans of confocal images of triple-labeled SCN tissue from rat were analyzed across rostro-to-caudal quadrants (Fig. 2). The pattern of little SAAS localization shifts from ventral in the rostral-most sections to central across the remaining quadrants. The little SAAS pattern matches that of GRP in the central SCN throughout middle and caudal planes. Merged pseudo-color images show some co-localization of little SAAS with VIP (pink) and GRP (yellow) (Fig. 2D, I, N, S, and Fig. S2, S3, S4 and S5). Regions of VIP/little SAAS overlap conform with the VIP sub-compartment that is extremely ventral in the rostral SCN (Fig. 2D), then medial within the middle and caudal SCN (Fig. 2H, L). This pattern of immunolocalization of VIP cells in the ventromedial SCN, although more medial than observed in some studies [6], [8], corroborates a previous report of two VIP neuronal subsets in the rat SCN [31]. GRP/little SAAS co-localization is relatively sparse rostrally and then increases to high density in the central SCN in the caudo-middle section, and more dorso-lateral in the caudal quadrant. Punctate expression, suggestive of pre-synaptic localization, also is observed. Overall, little SAAS colocalizes within the major cytoarchitectonic sub-compartments of VIP and GRP, appearing most intense in the central area demarcated by GRP.

### Volume and co-localization of little SAAS *vs.* VIP and GRP cell populations

We undertook stereological evaluation of little SAAS-, VIP- and GRP-expressing cells throughout the SCN. Little SAAS staining surrounds the dorsal SCN border. This pattern of little SAAS staining was evaluated as a marker delimiting the SCN. The Nissl-stained SCN, the standard marker of total SCN tissue, was compared to the extra-SCN region defined by dark staining for little SAAS. Total SCN volume was calculated based on SCN area in 40-µm serial coronal sections throughout the rostro-caudal axis. These SCN volume estimates were normalized to volume calculations based on Nissl staining, and show no significant difference between the volume prescribed by little SAAS *vs.* Nissl staining (*p*>0.95; data not shown).

Stereological analysis found an average of 1,636±247 little SAAS neurons (n = 6, based on 3 animals, two bins, counted per animal each containing 6 SCN sections at 120- µm intervals) for an estimated 16.4% of the ∼10,000 neurons in a single SCN. By comparison, VIP-expressing neurons (2,226±373; n = 3 animals, one bin counted) and GRP-expressing neurons (1,062±135; n = 3, one bin counted) account for 22.3% and 10.6% of the total number of neurons in a single SCN, respectively (Table 1). Neither GRP nor VIP neuron counts differ significantly compared to values previously reported for the rat SCN (*p*>0.12 and *p*>0.28, respectively, based on the Independent Groups T-test for Means) [8]. This assessment finds little SAAS-positive neurons to be a major peptidergic class, ranking after AVP and VIP and above GRP. Little SAAS is co-expressed with 10.4% of the VIP neurons (232±65; n = 3) and 79.2% of the GRP neurons (842±81; n = 3). Little SAAS is colocalized with GRP in ∼4× more neurons than with VIP, despite VIP-positive neurons being >2× more abundant than GRP-expressing cells. It is noteworthy that the total of little SAAS/VIP and little SAAS/GRP neurons accounts for only ∼66% of all little SAAS-positive neurons observed.

### Light induces c-FOS in little SAAS neurons

**Figure 3:**
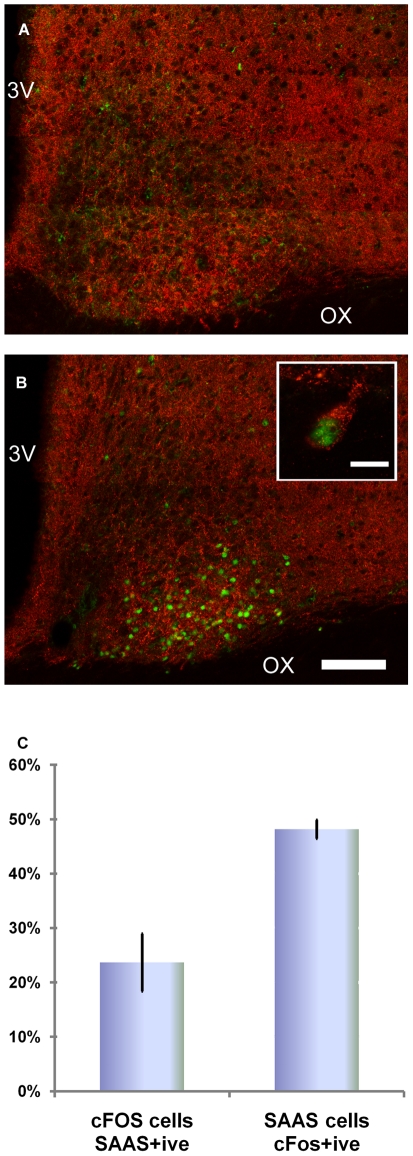
Light-induced c-FOS colocalizes with little SAAS in subset of neurons in the central SCN. (A–B) Confocal tile-scan images of SCN-containing coronal tissue sections from a rat receiving no light exposure *vs.* 400-lux light at CT 14 for 1 h. Light induction of c-FOS (green) is within the region of little SAAS staining (red) in the ventrolateral core SCN. High magnification optical section (z = 0.09 µm) reveals c-FOS (nuclear) and little SAAS (cytoplasmic) within the same cell. (C) Cell counting from mid-SCN sections found 23.7±5.3% of c-FOS-positive neurons also are little SAAS-positive, while 48.2±1.7% of little SAAS-positive neurons are also c-FOS positive. Tissue section thickness = 40 µm; optical image depth = 0.11 µm; 3V, third ventricle; OX, optic chiasm. Scale bar, A–B (shown in B) = 100 µm; scale bar, B inset = 10 µm.

Up-regulation of the immediate-early gene product, c-FOS, following light exposure during the dark phase of the circadian cycle is a hallmark of SCN activation: the intensity of c-FOS induction is proportional to the amplitude of the resulting change in phase of behavioral rhythms [32], [33]. Under stimulus conditions such as we used, which result in maximal delay, light-induced c-FOS expression is limited to a subset of neurons in the central SCN, implicating them in processing of the light signal [9], [34], [35]. In order to determine whether c-FOS is induced in little SAAS-positive neurons in response to light, rats were exposed to a light pulse at ZT 14 for 1 h and c-FOS localization was evaluated. Compared to controls (Fig. 3A), c-FOS immunoreactivity (green) overlaps with the region of dense little SAAS staining (red, Fig. 3B). Neurons expressing c-FOS/little SAAS comprise 23.7±5.3% of the total c-FOS-expressing neurons and 48.2±1.7% of little SAAS-positive neurons (n = 3; Fig. 3C). Thus, nearly 1/2 of little SAAS-positive neurons in the rat SCN are targets of the light signaling pathway, and ∼1/4 of the c-FOS-positive population express little SAAS neurons.

### Little SAAS is not expressed in the RHT

**Figure 4:**
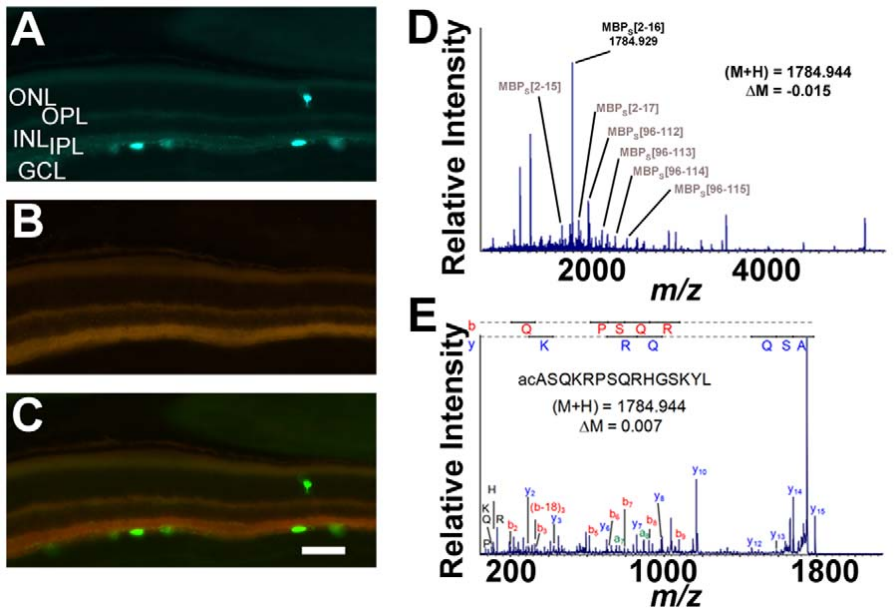
Little SAAS is not expressed in mouse melanopsin-positive retinal ganglion cells or rat optic nerve. (A–C) Confocal micrographs of male melanopsin-GFP transgenic mouse retina. (A) GFP-immunoreactivity (green) is localized to melanopsin-expressing cell bodies of the ganglion cell layer (GCL). (B) Little SAAS staining (red) is not observed above non-specific background levels in the same layer of retina. (C) Overlay of both channels shows no co-expression of little SAAS and melanopsin-GFP. (D–E) Mass spectrometry analysis of peptides in rat optic nerve. (D) Representative peptide profile of the optic nerve as detected by MALDI-TOF MS in extracts of individual nerve samples. Major peak at *m/z* 1784.9 corresponds to myelin basic protein (MBP_s_). Other truncated forms of MBP_S_ verified by tandem MS are labeled. (E) Tandem mass spectrometry by MALDI-TOF/TOF confirms identity of the 1784.9 peak as acetylated N-terminus fragment of MBP_S_. GCL, ganglion cell layer of the retina; INL, inner nuclear layer of the retina; IPL, inner plexiform layer of the retina; ME, median eminence; ONL, outer nuclear layer of the retina; OPL, outer plexiform layer of the retina; scale bar, C = 100 µm.

Because little SAAS is released from the horizontal SCN brain slice when the attached optic nerves innervating the SCN are stimulated, little SAAS could be a neurochemical messenger of retinohypothalamic tract (RHT) signaling from the eye. In order to assess whether little SAAS peptide is expressed in the light input pathway to the SCN, we examined little SAAS expression in retina and optic nerve. Melanopsin-containing retinal ganglion cells (RGCs) comprise 95% of the RGCs whose axons form the RHT within the optic nerve [36]. They are intrinsically photoreceptive elements of the circadian system and deliver light information directly to the SCN. Double immunohistochemistry of retinal tissue from the melanopsin-GFP transgenic mouse strongly immunolabeled melanopsin-GFP RGCs (green; Fig. 4A), but no little SAAS immunoreactivity above background was detected (red; Fig. 4B). Overlaying red and green channels confirms that melanopsin-GFP immunoreactivity does not colocalize little SAAS (Fig. 4C).

To evaluate little SAAS peptide content of optic nerve tissue from rat, we used matrix-assisted laser desorption/ionization-time of flight (MALDI-TOF) mass spectrometry (MS). These MS spectra reveal the presence of a peak at *m/z* 1784.93, which corresponds to the theoretical mass of the protonated ion for little SAAS (*m/z* 1784.97) (Fig. 4D). However, *de novo* sequencing by tandem MS performed on the 1784.9 peak identified it as acetylated N-terminus fragment of myelin basic protein, MBP_S_ (ac)ASQKRPSQRHGSKY, theoretical *m/z* = 1784.94 (Fig. 4E). The peptide exhibited a characteristic loss of CH_2_CO from the acetyl group as a result of metastable fragmentation when analyzed in reflectron mode. Numerous other MBP_s_ fragments were identified as well, thereby further confirming the origin of the 1784.9 peak in the ON extract analyzed. Thus, little SAAS is not present in either melanopsin-RGCs or optic nerve tissue in detectable amounts, and neither would be a source of little SAAS detected in releasate at the SCN upon optic nerve stimulation.

### Little SAAS acts downstream of NMDA-receptor signaling

**Figure 5:**
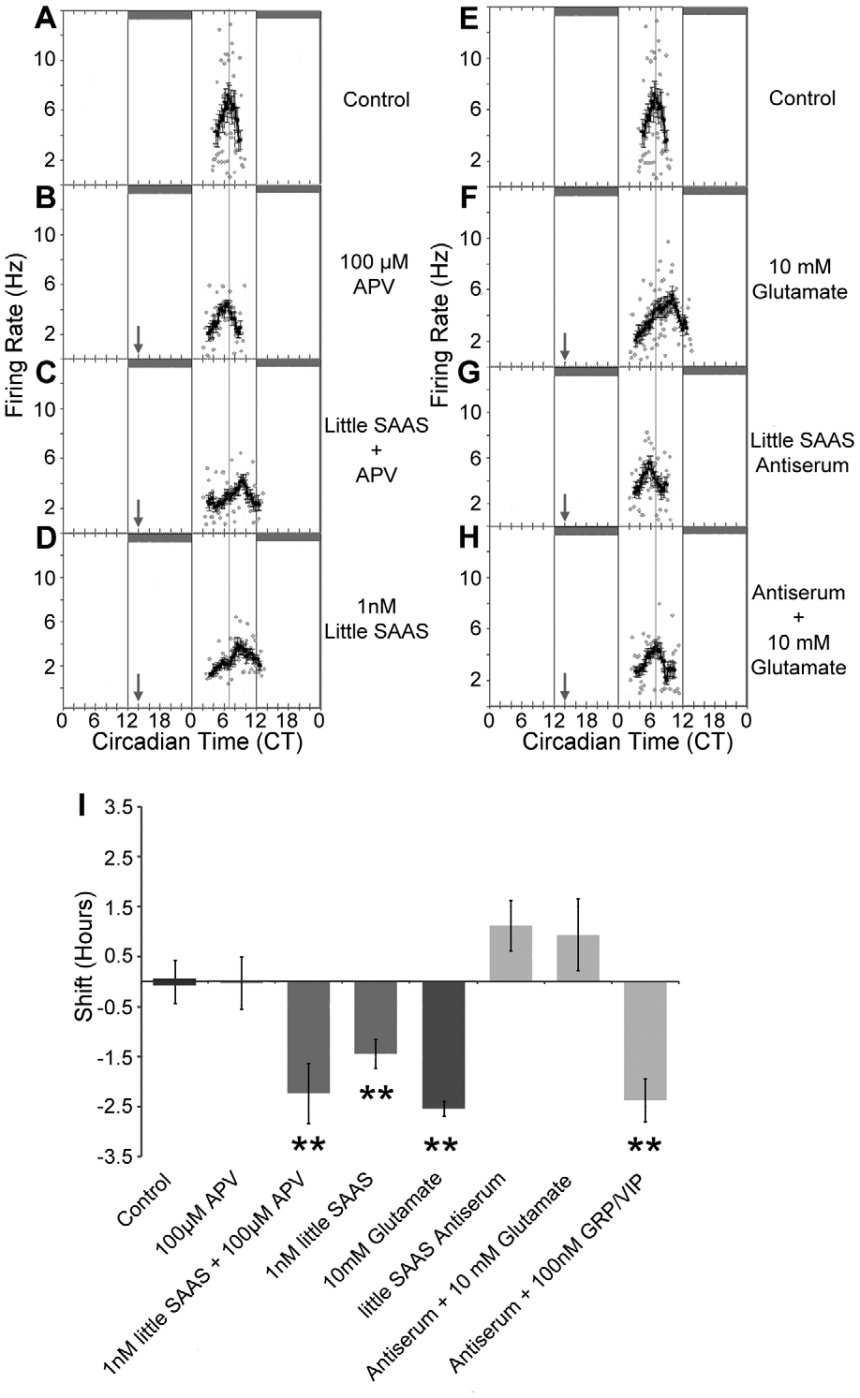
Little SAAS acts downstream of NMDAR and parallel to VIP/GRP signaling during early night. (A, I) SCN in a coronal brain slice displays characteristic mid-subjective daytime peak at CT 7 in spontaneous rhythm of neuronal firing rate. (B, I) The competitive NMDAR antagonist, APV (100 µM), does not alter phase. (C, I) Application of little SAAS following pre-treatment with APV results in phase delay. (D, I) Application of little SAAS alone causes a similar phase delay. (E, I) Firing rate rhythm in control SCN slice with peak at CT 7. (F, I) Application of glutamate (10 mM, 1µL drop) at CT 14 causes phase delay. (G, I) Application of little SAAS antiserum (#2768,undiluted) does not significantly alter SCN phasing. (H, I) Little SAAS antiserum blocks the phase delay induced by glutamate. (I) Mean data demonstrate that whereas the NMDAR antagonist, APV, does not block little SAAS action, little SAAS antiserum blocks the phase delaying effect of glutamate, but does not affect not the phase delay stimulated by the GRP/VIP cocktail. n≥3 for all groups; **, *p*≤0.001 *vs.* control, one-way ANOVA with Bonferroni post-hoc test.

The primary mechanism by which the RHT communicates phase delay is glutamate signaling via NMDA receptor (NMDAR) activation, an event that is both necessary and sufficient to shift SCN phase [24]. To evaluate whether little SAAS functions downstream of the NMDAR, the competitive NMDAR antagonist, (2*R*)-amino-5-phosphonovaleric acid (APV; 1 µL drop/SCN, 100 µM), was applied to the SCN brain slice 10 min prior to application of little SAAS (1 µL drop/SCN, 1 nM) at CT14. APV alone does not alter clock timing (−0.04±0.52 h; n = 3; Fig. 5B, I). Further, APV does not alter the little SAAS-induced phase delay (−2.25±0.60 h; n = 4; Fig. 5C, I).

To assess whether little SAAS is required for intercellular communication within the SCN during glutamatergic phase delay, the SCN was pre-incubated (10 min) with a 1 µL drop of undiluted little SAAS antiserum and then treated with glutamate (1 µL drop, 10 mM), both applied directly to the SCN in the brain slice. Little SAAS antiserum alone does not alter SCN phase (Fig. 5G, I). When applied prior to glutamate at CT 14, little SAAS antiserum blocks glutamate-induced phase delay (0.94±0.72 h; n = 4; Fig. 5H, I) compared to glutamate alone (−2.54±0.14 h; n = 3; p≤0.01, one-way ANOVA with Bonferroni post-hoc test; Fig. 5F, I). Further, little SAAS antiserum does not block phase delay caused by a cocktail of VIP and GRP (−2.38±0.43 h; n = 3; p≤0.01, one-way ANOVA with Bonferroni post-hoc test; Fig. 5I).

### Little SAAS signaling is independent of VPAC_2_R/BB_2_R signaling

**Figure 6:**
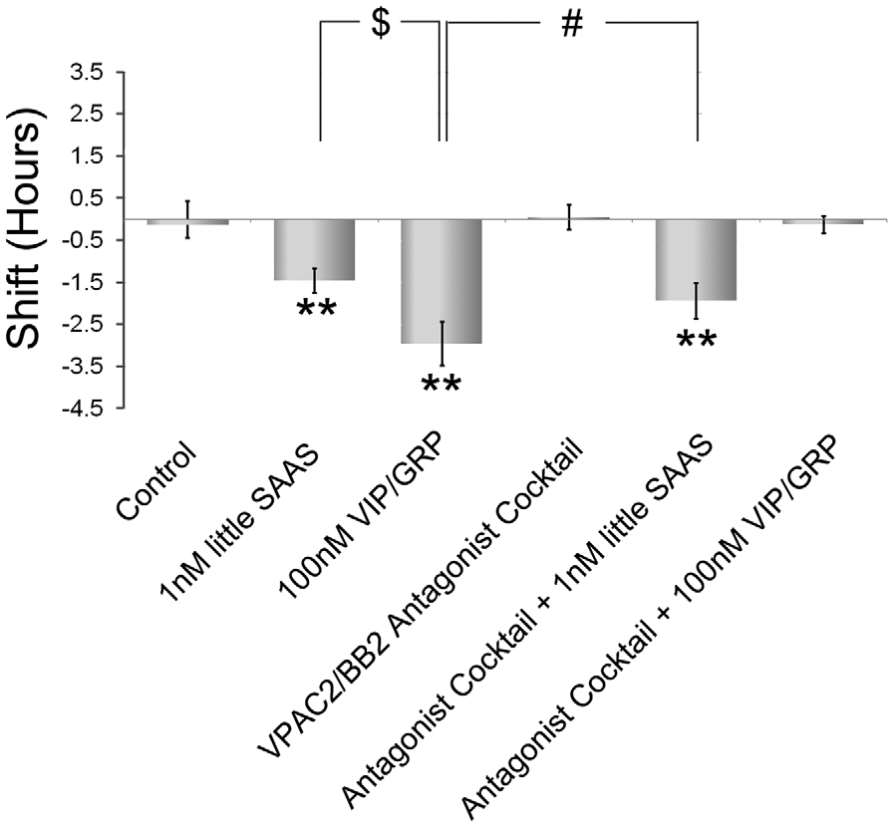
Effects of little SAAS on SCN phasing are independent of VPAC2/BB2 signaling. Microdrop application to the SCN at CT 14 of little SAAS (1 or 100 nM) or VIP/GRP (100 nM each) induces significant phase delay compared with controls (*p*≤0.01). Pre-incubation with a cocktail of VPAC2 receptor antagonist [4Cl-D-Phe,Leu]VIP (50 mM)/BB2 receptor antagonist PD176252 (50 mM) does not alter SCN phasing (0.04±0.29 h) *vs.* controls. The antagonist cocktail does not alter the phase delay induced by little SAAS (−1.94±0.43) but fully blocks the phase shift induced by a VIP/GRP (−0.13±0.20 h). n≥3 for all groups; **, *p*≤0.01 *vs.* control; $, *p*≤0.01 *vs.* 100 nM VIP/GRP cocktail; #, *p*≤0.05 *vs.* 100 nM VIP/GRP cocktail, one-way ANOVA w/Tukey-Kramer post-hoc test.

VIP and GRP, acting via their respective G protein-coupled receptors, VPAC_2_R and BB_2_R, respectively, mediate intra-SCN communication driving synchronous phase shifting of the SCN [11], [17]. To evaluate whether VPAC_2_R and BB_2_R signaling is downstream of little SAAS phase delay at CT14, a cocktail containing selective antagonists for VPAC_2_R ([4Cl-D-Phe^6^, Leu^17^] VIP, 50mM) [37], [38], [39] and BB_2_R (PD176252, 50mM) [40], [41] was used (Fig. 6). The antagonist cocktail (50 mM each in 1 µL/SCN drop/10 min) does not affect clock phase (0.04±0.29 h; n = 3). Whereas application of a VIP/GRP cocktail (100 µM each in 1 µL) causes the anticipated delay in clock phase (−2.96±0.52 h; n = 3; *p*≤0.01, one-way ANOVA with Tukey-Kramer post-test), this effect is blocked fully by pre-incubation with the VPAC_2_R/BB_2_R antagonist cocktail (0.13±0.20 h; n = 4). Pre-treatment with this antagonist cocktail (1 µM in1 µL drop) has no effect on the amplitude of phase delay caused by little SAAS (−1.94±0.43 h; n = 4; *p*≤0.01, one-way ANOVA with Tukey-Kramer post-hoc test).

## Discussion

This study evaluates the localization and functional context of little SAAS within neuropeptidergic circuits of the SCN. We find that little SAAS is expressed in the central sub-compartment of rat and mouse, but is not detected in either SCN-projecting melanopsin RGCs of the retina or optic nerve tissue. Triple immunohistochemistry for VIP, GRP and little SAAS reveals a combination of single- and multi-peptide-expressing cellular phenotypes that colocalize most densely within the central SCN. Little SAAS is a major class of peptide-expressing neurons within the SCN, more abundant than GRP. About 50% of little SAAS neurons respond to light exposure in early nighttime with induction of c-FOS. The partial response of little SAAS neurons to light with c-FOS induction may indicate that the neurons not responding participate in integrating distinct features of the stimulus coming from the retina. Alternatively, part of the little SAAS subpopulation may have an entirely different role from the light-responders, such as integrating input from the thalamus or raphe.

**Figure 7:**
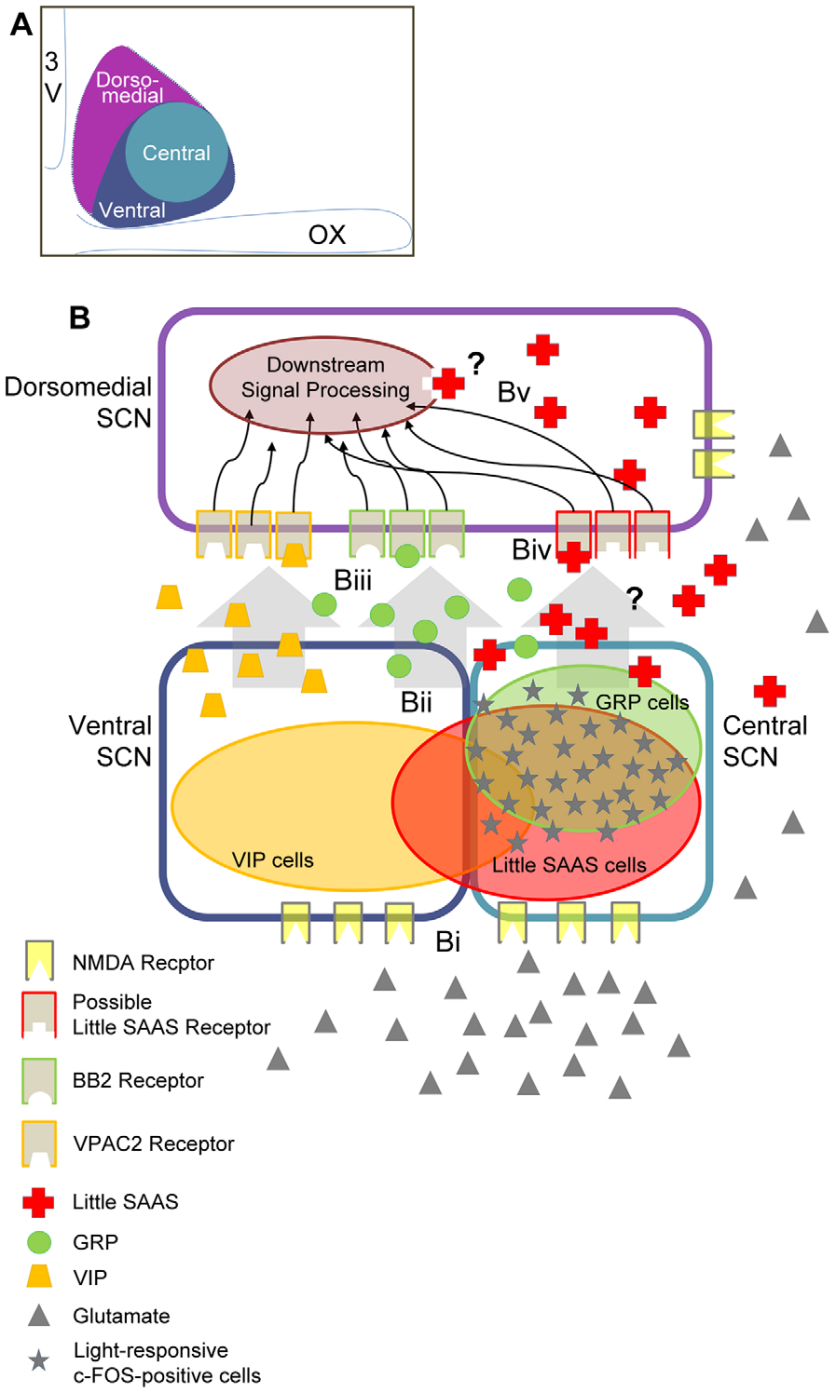
Model of light signal processing in the rat SCN. (A) Schematic of general functional organization within the rat SCN (coronal section, mid-SCN). SCN regions (ventral, dark blue; central, light blue; and dorsomedial, purple) denote functionally distinct regions that receive and integrate different signals (central/ventral) and relay this information locally and to the dorsomedial SCN. These regions also extend efferents to nearby hypothalamus. (B) Peptidergic signal processing within the SCN. (Bi) Glutamate released from RHT terminals activates NMDA receptors, which are expressed on neurons throughout the SCN [64] (Bii) Signal transduction downstream of NMDAR activation leads to c-FOS induction and peptide release in a subset of neurons. Intra-SCN signaling is mediated by VIP, GRP and/or little SAAS peptides. (Biii) VIP and GRP transmit signals via VPAC2 and BB2 receptors, respectively. Little SAAS functions independently of VPAC2 or BB2 receptor signaling, and is predicted to function through (Biv) an unidentified cell surface receptor, and (Bv) downstream signal-processing events.

Exogenous little SAAS peptide applied to the SCN brain slice during early subjective nighttime (CT 14) elicits phase delay of the SCN rhythm of neuronal firing rate. Pharmacological blockade of the NMDA receptor does not alter the ability of little SAAS to cause phase delay, nor does inhibition of VPAC_2_/BB_2_ receptors. This places little SAAS as a signaling element in an intra-SCN circuit, downstream of NMDAR activation and distinct from VIP/GRP signaling (Fig. 7).

This study defines the functional context of a new SCN peptide discovered by forward peptidomics. It builds upon direct mass spectrometric identification of a molecular species, but relies on the specificity of antisera, which are indirect reagents. Thus, demonstration of antisera specificity is critical. The little SAAS antisera used in this study are highly specific; patterned immunoreactivity in the wild-type mouse brain is similar to rat, and is absent in the brain of the proSAAS null mouse. Additionally, pre-absorption of little SAAS antibodies with little SAAS peptide or application of only the secondary antibody does not result in immunostaining of rat hypothalamic tissue. One of the little SAAS antisera used in this study (#2766) previously has been reported to strongly recognize its immunogenic peptide, little SAAS (proSAAS-(42–59)), as well as big SAAS (proSAAS-(34–59)), but not the KEP peptide (proSAAS-(34–40)) [27]. The same antiserum also has demonstrated some affinity for synthetic full-length proSAAS. Peptidomic studies of SON and SCN tissue extracts identified a number of truncated forms of little SAAS [3], [42]; however, only little SAAS (proSAAS-(42–59)) has been detected in releasates from the rat SCN under physiological conditions [7], emphasizing that little SAAS (proSAAS-(42–59)) is likely a physiological form of this peptide.

The discrete localization of little SAAS within the central SCN predicts a role in processing afferent signals. This region receives light information through direct projections from the eye through the RHT. Axons of melanopsin RGCs that form the RHT colocalize glutamate and PACAP in terminals that invest the SCN [43], [44], [45]. Little SAAS action in the SCN does not originate from direct retinal projections; this is indicated by absence of either little SAAS immunoreactivity in the melanopsin RGCs of mouse or bona fide little SAAS peptide determined by mass spectrometry of the ON of rat. Rather, little SAAS may be entirely endogenous to the SCN or some could be released from non-RHT afferents.

The central and ventromedial regions of the SCN also receive indirect projections from the eye through the geniculohypothalamic tract [6], [10]. In addition, there is overlapping innervation from serotonergic projections from the median raphe. These non-photic pathways have been implicated in generating mid-day phase advances and attenuating glutamatergic, light-stimulated phase shifts during the nighttime [46], [47], [48], [49]. Convergence of multiple afferents on overlapping topographical targets provides strong evidence that neurons there comprise local circuits through which the SCN integrates and relays afferent signals reporting environmental or organismal change.

VIP- and GRP-expressing neurons are integral components of the SCN circuitry that synchronizes cellular clocks and generates coherent light-induced resetting of clock phase [50], [51], [52], [53], [54], [55], [56], [57]. Both peptidergic populations extend collateral processes locally, as well as to the dorsal AVP-containing region [58]. Either singly or together as a cocktail, VIP and GRP are effective activators of light-like resetting of behavioral rhythms [59], [60]. The action of VIP and GRP on phase shifting is conveyed via the VPAC2 and BB2 receptors, respectively. Activation of these receptors has been linked to propagating the induction of *Period* genes within the SCN during the light response of mouse and rat [52], [61]. These findings suggest that VIP and GRP may interact at the circuit level to achieve a wide range of responses.

Co-localization of little SAAS with subsets of VIP and GRP neurons in the central sub-compartment suggests that little SAAS also could participate in signal integration and relay within SCN circuits (Fig. 7). This possibility is supported by the relatively large percentage of SCN neurons containing little SAAS. Stereological examination finds that little SAAS is the third most abundant peptidergic class, after AVP and VIP, identified to date in the rat SCN [4]. Whereas only ∼10% of VIP neurons are little SAAS-positive, nearly 80% of GRP neurons co-localize little SAAS. Moreover, the total number of little SAAS-positive neurons is higher than the number of little SAAS/VIP and little SAAS/GRP co-expressing neurons. These data support the conjecture that little SAAS marks a functionally important peptidergic subpopulation that is not accounted for by previously defined SCN neurons.

Our findings support a role for little SAAS in the signal integration and relay circuitry that mediates the circadian light response. c-FOS induction in central and core SCN neurons is an established marker of light activation. In the mouse SCN, roughly 70% of the GRP-expressing neurons exhibit light responsive c-FOS induction [9]. In our assessment, approximately 50% of little SAAS neurons in rat express c-FOS following light pulse. Thus, despite species differences, light-responsive little SAAS and GRP neurons are similar numerically to the number of little SAAS/GRP co-expressing neurons determined through our stereological analysis. Nevertheless, the NMDA antagonist, AP-5, which effectively blocks light-like phase shifts caused by glutamate or NMDA [62], fails to block the phase-shifting effects of little SAAS. This is in contrast to the finding that NMDA antagonists do block the phase shifting effects of GRP [63]. This further distinguishes little SAAS from the other SCN neuropeptides. It is noteworthy that little SAAS and GRP have such a high co-localization and yet have apparently differing relationships to NMDA-dependent signal transduction in the SCN. Taken together, these data raise questions as to whether little SAAS/GRP co-expressing neurons have a specific role in the SCN response to light, a finding that warrants investigation.

The light responsiveness of little SAAS neurons, combined with the insensitivity of the little-SAAS phase shift to blockade of VIP and GRP receptors, tentatively places little SAAS in SCN circuitry downstream of glutamate/NMDAR signaling and parallel to VIP/GRP (Fig. 7). Previously, we determined that exogenous little SAAS is capable of shifting the phase of the circadian clock in the SCN brain slice during early night, similar to phase delays evoked by VIP and GRP [7], [53], [54]. Little SAAS release from the SCN slice is low during subjective nighttime [7], coinciding with high SCN sensitivity to little SAAS. The possibility that endogenous little SAAS levels might modulate SCN susceptibility to phase resetting deserves further evaluation.

Overall, this study has built upon discovery-based peptidomics to localize and functionally define a context for little SAAS in the neuropeptidergic circuitry of the SCN. This role for little SAAS is independent of previously described VIP and GRP signal relay, positioning little SAAS as a third element in intra-SCN cell-to-cell communication that mediates resetting of the circadian clock. This finding further implicates SCN neuropeptidergic heterogeneity in multi-factorial communication of circadian timekeeping and time-resetting. While VIP, GRP and little SAAS influence clock phase shifting in similar ways, different combinations of stimulus type, clock state and physiological variables may evoke different contextual roles for these neuropeptides in SCN phase regulation *in vivo*. The emerging, unanticipated complexity of SCN neuropeptide actions emphasizes the need to re-evaluate their multivariate roles in SCN physiology.

## Supporting Information

Figure S1 Specificity of little SAAS antiserum. In order to evaluate the little SAAS antiserum used in this study, two stringent tests were performed. The first evaluated SCN immunoreactivity in transgenic proSAAS-null mice compared with wild types. Little SAAS staining within the brain tissue of the wild-type mouse (S1A) is absent in the proSAAS-null mouse tissue (S1B). Similar results are observed with both antisera #2766 and 2768 against little SAAS (S1 shows antiserum #2766). The second test evaluated staining in rat tissue using antisera pre-incubated in 100 µM little SAAS peptide. When either little SAAS antisera was pre-absorbed, no little SAAS staining is observed in rat brain (data not shown). These data validate the specificity of these antisera for little SAAS. Scale bar = 1 mm.
(1.36 MB TIF)

Figure S2 Enlarged confocal image of VIP/GRP/little SAAS immunostained rat rostral SCN. Enlargement of Figure 2D reveals expression of respective peptides in this quadrant of the rat SCN: VIP (white), GRP (green), little SAAS (red), VIP/little SAAS (pink), and GRP/little SAAS (yellow). 3V, third ventricle; OX, optic chiasm. Single optical section, z = 0.11 µm. Scale bar = 100 µm.
(9.47 MB TIF)

Figure S3 Enlarged confocal image of VIP/GRP/little SAAS immunostained rat rostro-medial SCN. Enlargement of Figure 2I reveals expression of respective peptides in this quadrant of the rat SCN: VIP (white), GRP (green), little SAAS (red), VIP/little SAAS (pink), and GRP/little SAAS (yellow). 3V, third ventricle; OX, optic chiasm. Single optical section, z = 0.11 µm. Scale bar = 100 µm.
(9.99 MB TIF)

Figure S4 Enlarged confocal image of VIP/GRP/little SAAS immunostained rat medio-caudal SCN. Enlargement of Figure 2N reveals expression of respective peptides in this quadrant of the rat SCN: VIP (white), GRP (green), little SAAS (red), VIP/little SAAS (pink), and GRP/little SAAS (yellow). 3V, third ventricle; OX, optic chiasm. Single optical section, z = 0.11 µm. Scale bar = 100 µm.
(9.39 MB TIF)

Figure S5 Enlarged confocal image of VIP/GRP/little SAAS immunostained rat caudal SCN. Enlargement of Figure 2S reveals expression of respective peptides in this quadrant of the rat SCN: VIP (white), GRP (green), little SAAS (red), VIP/little SAAS (pink), and GRP/little SAAS (yellow). 3V, third ventricle; OX, optic chiasm. Single optical section, z = 0.11 µm. Scale bar = 100 µm.
(9.80 MB TIF)

## References

[1] Davidson AJ, Yamazaki S, Menaker M (2003). SCN: ringmaster of the circadian circus or conductor of the circadian orchestra?. Novartis Found Symp.

[2] Gillette MU, Sejnowski TJ (2005). Physiology. Biological clocks coordinately keep life on time.. Science.

[3] Lee JE, Atkins N, Hatcher NG, Zamdborg L, Gillette MU (2010). Endogenous peptide discovery of the rat circadian clock: a focused study of the suprachiasmatic nucleus by ultra-high performance tandem mass spectrometry.. Mol Cell Proteomics.

[4] van den Pol AN (1980). The hypothalamic suprachiasmatic nucleus of rat: intrinsic anatomy.. J Comp Neurol.

[5] Morin LP, Shivers KY, Blanchard JH, Muscat L (2006). Complex organization of mouse and rat suprachiasmatic nucleus.. Neuroscience.

[6] Abrahamson EE, Moore RY (2001). Suprachiasmatic nucleus in the mouse: retinal innervation, intrinsic organization and efferent projections.. Brain Res.

[7] Hatcher NG, Atkins N, Annangudi SP, Forbes AJ, Kelleher NL (2008). Mass spectrometry-based discovery of circadian peptides.. Proc Natl Acad Sci U S A.

[8] Moore RY, Speh JC, Leak RK (2002). Suprachiasmatic nucleus organization.. Cell Tissue Res.

[9] Karatsoreos IN, Yan L, LeSauter J, Silver R (2004). Phenotype matters: identification of light-responsive cells in the mouse suprachiasmatic nucleus.. J Neurosci.

[10] Morin LP, Allen CN (2006). The circadian visual system, 2005.. Brain Res Rev.

[11] Yan L, Karatsoreos I, Lesauter J, Welsh DK, Kay S (2007). Exploring spatiotemporal organization of SCN circuits.. Cold Spring Harb Symp Quant Biol.

[12] Morin LP (2007). SCN organization reconsidered.. J Biol Rhythms.

[13] Cassone VM, Speh JC, Card JP, Moore RY (1988). Comparative anatomy of the mammalian hypothalamic suprachiasmatic nucleus.. J Biol Rhythms.

[14] Card JP, Moore RY (1984). The suprachiasmatic nucleus of the golden hamster: immunohistochemical analysis of cell and fiber distribution.. Neuroscience.

[15] Antle MC, Silver R (2005). Orchestrating time: arrangements of the brain circadian clock.. Trends Neurosci.

[16] Jobst EE, Allen CN (2002). Calbindin neurons in the hamster suprachiasmatic nucleus do not exhibit a circadian variation in spontaneous firing rate.. Eur J Neurosci.

[17] Harmar AJ (2003). An essential role for peptidergic signalling in the control of circadian rhythms in the suprachiasmatic nuclei.. J Neuroendocrinol.

[18] Fricker LD, McKinzie AA, Sun J, Curran E, Qian Y (2000). Identification and characterization of proSAAS, a granin-like neuroendocrine peptide precursor that inhibits prohormone processing.. J Neurosci.

[19] Gouraud SS, Heesom K, Yao ST, Qiu J, Paton JF (2007). Dehydration-induced proteome changes in the rat hypothalamo-neurohypophyseal system.. Endocrinology.

[20] Tischkau SA, Mitchell JW, Pace LA, Barnes JW, Barnes JA (2004). Protein kinase G type II is required for night-to-day progression of the mammalian circadian clock.. Neuron.

[21] Prosser RA, Gillette MU (1989). The mammalian circadian clock in the suprachiasmatic nuclei is reset in vitro by cAMP.. J Neurosci.

[22] Novak A, Guo C, Yang W, Nagy A, Lobe CG (2000). Z/EG, a double reporter mouse line that expresses enhanced green fluorescent protein upon Cre-mediated excision.. Genesis.

[23] Morgan DJ, Wei S, Gomes I, Czyzyk T, Mzhavia N (2010). The propeptide precursor proSAAS is involved in fetal neuropeptide processing and body weight regulation.. J Neurochem.

[24] Ding JM, Chen D, Weber ET, Faiman LE, Rea MA (1994). Resetting the biological clock: mediation of nocturnal circadian shifts by glutamate and NO.. Science.

[25] Gillette MU (1986). The suprachiasmatic nuclei: circadian phase-shifts induced at the time of hypothalamic slice preparation are preserved in vitro.. Brain Res.

[26] Gillette MU, Medanic M, McArthur AJ, Liu C, Ding JM (1995). Intrinsic neuronal rhythms in the suprachiasmatic nuclei and their adjustment.. Ciba Found Symp.

[27] Mzhavia N, Berman Y, Che FY, Fricker LD, Devi LA (2001). ProSAAS processing in mouse brain and pituitary.. J Biol Chem.

[28] Beaulé C, Mitchell JW, Lindberg PT, Damadzic R, Eiden LE (2009). Temporally restricted role of retinal PACAP: integration of the phase-advancing light signal to the SCN.. J Biol Rhythms.

[29] Karatsoreos IN, Wang A, Sasanian J, Silver R (2007). A role for androgens in regulating circadian behavior and the suprachiasmatic nucleus.. Endocrinology.

[30] Romanova EV, Rubakhin SS, Sweedler JV (2008). One-step sampling, extraction, and storage protocol for peptidomics using dihydroxybenzoic Acid.. Anal Chem.

[31] Kawamoto K, Nagano M, Kanda F, Chihara K, Shigeyoshi Y (2003). Two types of VIP neuronal components in rat suprachiasmatic nucleus.. J Neurosci Res.

[32] Beaule C, Arvanitogiannis A, Amir S (2001). Light suppresses Fos expression in the shell region of the suprachiasmatic nucleus at dusk and dawn: implications for photic entrainment of circadian rhythms.. Neuroscience.

[33] Kornhauser JM, Nelson DE, Mayo KE, Takahashi JS (1990). Photic and circadian regulation of c-fos gene expression in the hamster suprachiasmatic nucleus.. Neuron.

[34] Silver R, Romero MT, Besmer HR, Leak R, Nunez JM (1996). Calbindin-D28K cells in the hamster SCN express light-induced Fos.. Neuroreport.

[35] Edelstein K, Beaule C, D'Abramo R, Amir S (2000). Expression profiles of JunB and c-Fos proteins in the rat circadian system.. Brain Res.

[36] Hattar S, Kumar M, Park A, Tong P, Tung J (2006). Central projections of melanopsin-expressing retinal ganglion cells in the mouse.. J Comp Neurol.

[37] Pandol SJ, Dharmsathaphorn K, Schoeffield MS, Vale W, Rivier J (1986). Vasoactive intestinal peptide receptor antagonist [4Cl-D-Phe6, Leu17] VIP.. Am J Physiol.

[38] Weick RF, Stobie KM (1995). Role of VIP in the regulation of LH secretion in the female rat.. Neurosci Biobehav Rev.

[39] Weick RF, Stobie KM, Noh KA (1992). Effect of [4Cl-D-Phe6,Leu17]VIP on the inhibition of pulsatile LH release by VIP and related peptides in the ovariectomized rat.. Neuroendocrinology.

[40] Ashwood V, Brownhill V, Higginbottom M, Horwell DC, Hughes J (1998). PD 176252–the first high affinity non-peptide gastrin-releasing peptide (BB2) receptor antagonist.. Bioorg Med Chem Lett.

[41] Moody TW, Nakagawa T, Kang Y, Jakowlew S, Chan D (2006). Bombesin/gastrin-releasing peptide receptor antagonists increase the ability of histone deacetylase inhibitors to reduce lung cancer proliferation.. J Mol Neurosci.

[42] Bora A, Annangudi SP, Millet LJ, Rubakhin SS, Forbes AJ (2008). Neuropeptidomics of the supraoptic rat nucleus.. J Proteome Res.

[43] Gooley JJ, Lu J, Chou TC, Scammell TE, Saper CB (2001). Melanopsin in cells of origin of the retinohypothalamic tract.. Nat Neurosci.

[44] Hannibal J, Ding JM, Chen D, Fahrenkrug J, Larsen PJ (1997). Pituitary adenylate cyclase-activating peptide (PACAP) in the retinohypothalamic tract: a potential daytime regulator of the biological clock.. J Neurosci.

[45] Hannibal J, Fahrenkrug J (2002). Melanopsin: a novel photopigment involved in the photoentrainment of the brain's biological clock?. Ann Med.

[46] Bradbury MJ, Dement WC, Edgar DM (1997). Serotonin-containing fibers in the suprachiasmatic hypothalamus attenuate light-induced phase delays in mice.. Brain Res.

[47] Edgar DM, Miller JD, Prosser RA, Dean RR, Dement WC (1993). Serotonin and the mammalian circadian system: II. Phase-shifting rat behavioral rhythms with serotonergic agonists.. J Biol Rhythms.

[48] Edgar DM, Reid MS, Dement WC (1997). Serotonergic afferents mediate activity-dependent entrainment of the mouse circadian clock.. Am J Physiol.

[49] Medanic M, Gillette MU (1992). Serotonin regulates the phase of the rat suprachiasmatic circadian pacemaker in vitro only during the subjective day.. J Physiol.

[50] Aton SJ, Colwell CS, Harmar AJ, Waschek J, Herzog ED (2005). Vasoactive intestinal polypeptide mediates circadian rhythmicity and synchrony in mammalian clock neurons.. Nat Neurosci.

[51] Isobe Y, Nishino H (1996). Vasoactive intestinal peptide and gastrin-releasing peptide play distinct roles in the suprachiasmatic nucleus.. Brain Res Bull.

[52] Nielsen HS, Hannibal J, Fahrenkrug J (2002). Vasoactive intestinal polypeptide induces per1 and per2 gene expression in the rat suprachiasmatic nucleus late at night.. Eur J Neurosci.

[53] McArthur AJ, Coogan AN, Ajpru S, Sugden D, Biello SM (2000). Gastrin-releasing peptide phase-shifts suprachiasmatic nuclei neuronal rhythms in vitro.. J Neurosci.

[54] Reed HE, Meyer-Spasche A, Cutler DJ, Coen CW, Piggins HD (2001). Vasoactive intestinal polypeptide (VIP) phase-shifts the rat suprachiasmatic nucleus clock in vitro.. Eur J Neurosci.

[55] Romijn HJ, Sluiter AA, Pool CW, Wortel J, Buijs RM (1996). Differences in colocalization between Fos and PHI, GRP, VIP and VP in neurons of the rat suprachiasmatic nucleus after a light stimulus during the phase delay versus the phase advance period of the night.. J Comp Neurol.

[56] Romijn HJ, Sluiter AA, Wortel J, Van Uum JF, Buijs RM (1998). Immunocytochemical evidence for a diurnal rhythm of neurons showing colocalization of VIP with GRP in the rat suprachiasmatic nucleus.. J Comp Neurol.

[57] Tanaka M, Matsuda T, Shigeyoshi Y, Ibata Y, Okamura H (1997). Peptide expression in GABAergic neurons in rat suprachiasmatic nucleus in comparison with other forebrain structures: a double labeling in situ hybridization study.. J Histochem Cytochem.

[58] Romijn HJ, Sluiter AA, Pool CW, Wortel J, Buijs RM (1997). Evidence from confocal fluorescence microscopy for a dense, reciprocal innervation between AVP-, somatostatin-, VIP/PHI-, GRP-, and VIP/PHI/GRP-immunoreactive neurons in the rat suprachiasmatic nucleus.. Eur J Neurosci.

[59] Albers HE, Liou SY, Stopa EG, Zoeller RT (1991). Interaction of colocalized neuropeptides: functional significance in the circadian timing system.. J Neurosci.

[60] Albers HE, Gillespie CF, Babagbemi TO, Huhman KL (1995). Analysis of the phase shifting effects of gastrin releasing peptide when microinjected into the suprachiasmatic region.. Neurosci Lett.

[61] Aida R, Moriya T, Araki M, Akiyama M, Wada K (2002). Gastrin-releasing peptide mediates photic entrainable signals to dorsal subsets of suprachiasmatic nucleus via induction of Period gene in mice.. Mol Pharmacol.

[62] Mintz EM, Marvel CL, Gillespie CF, Price KM, Albers HE (1999). Activation of NMDA receptors in the suprachiasmatic nucleus produces light-like phase shifts of the circadian clock in vivo.. J Neurosci.

[63] Kallingal GJ, Mintz EM (2006). Glutamatergic activity modulates the phase-shifting effects of gastrin-releasing peptide and light.. Eur J Neurosci.

[64] Stamp JA, Piggins HD, Rusak B, Semba K (1997). Distribution of ionotropic glutamate receptor subunit immunoreactivity in the suprachiasmatic nucleus and intergeniculate leaflet of the hamster.. Brain Res.

