# Supplementary figures and images for: Circadian Integration of Glutamatergic Signals by Little SAAS in Novel Suprachiasmatic Circuits
Source: PLoS One. 2010 Sep 7;5(9):e12612. doi: 10.1371/journal.pone.0012612 (PMC2935382; doi:10.1371/journal.pone.0012612)

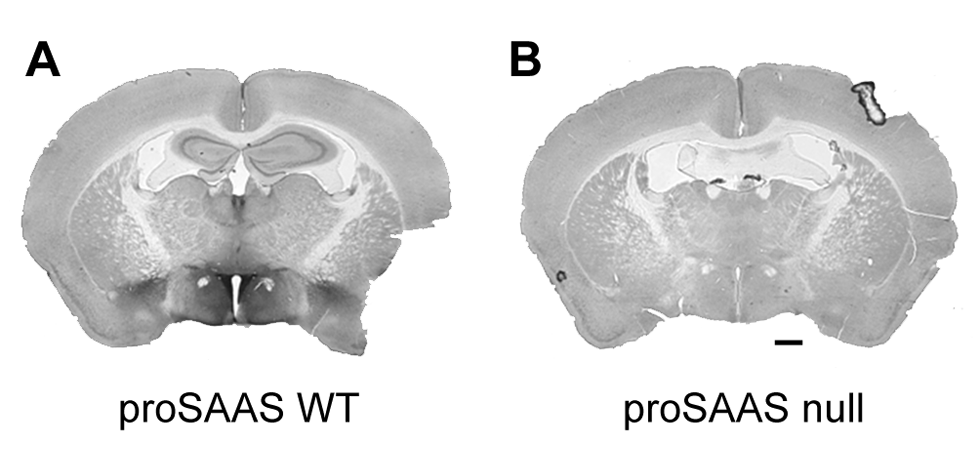

Supplement: Figure S1 — Specificity of little SAAS antiserum. In order to evaluate the little SAAS antiserum used in this study, two stringent tests were performed. The first evaluated SCN immunoreactivity in transgenic proSAAS-null mice compared with wild types. Little SAAS staining within the brain tissue of the wild-type mouse (S1A) is absent in the proSAAS-null mouse tissue (S1B). Similar results are observed with both antisera #2766 and 2768 against little SAAS (S1 shows antiserum #2766). The second test evaluated staining in rat tissue using antisera pre-incubated in 100 µM little SAAS peptide. When either little SAAS antisera was pre-absorbed, no little SAAS staining is observed in rat brain (data not shown). These data validate the specificity of these antisera for little SAAS. Scale bar = 1 mm. (1.36 MB TIF) [file pone.0012612.s001.tif]

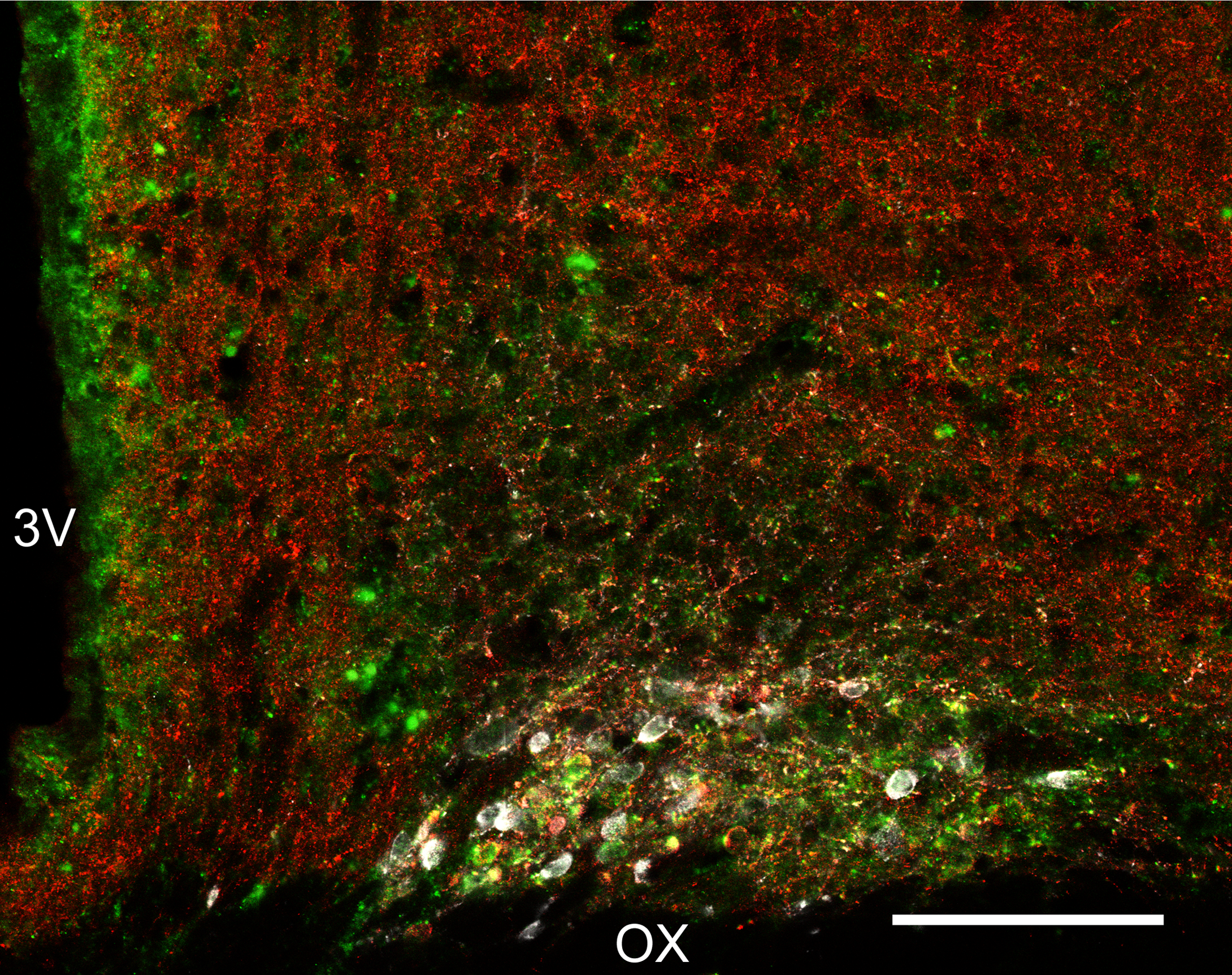

Supplement: Figure S2 — Enlarged confocal image of VIP/GRP/little SAAS immunostained rat rostral SCN. Enlargement of Figure 2D reveals expression of respective peptides in this quadrant of the rat SCN: VIP (white), GRP (green), little SAAS (red), VIP/little SAAS (pink), and GRP/little SAAS (yellow). 3V, third ventricle; OX, optic chiasm. Single optical section, z = 0.11 µm. Scale bar = 100 µm. (9.47 MB TIF) [file pone.0012612.s002.tif]

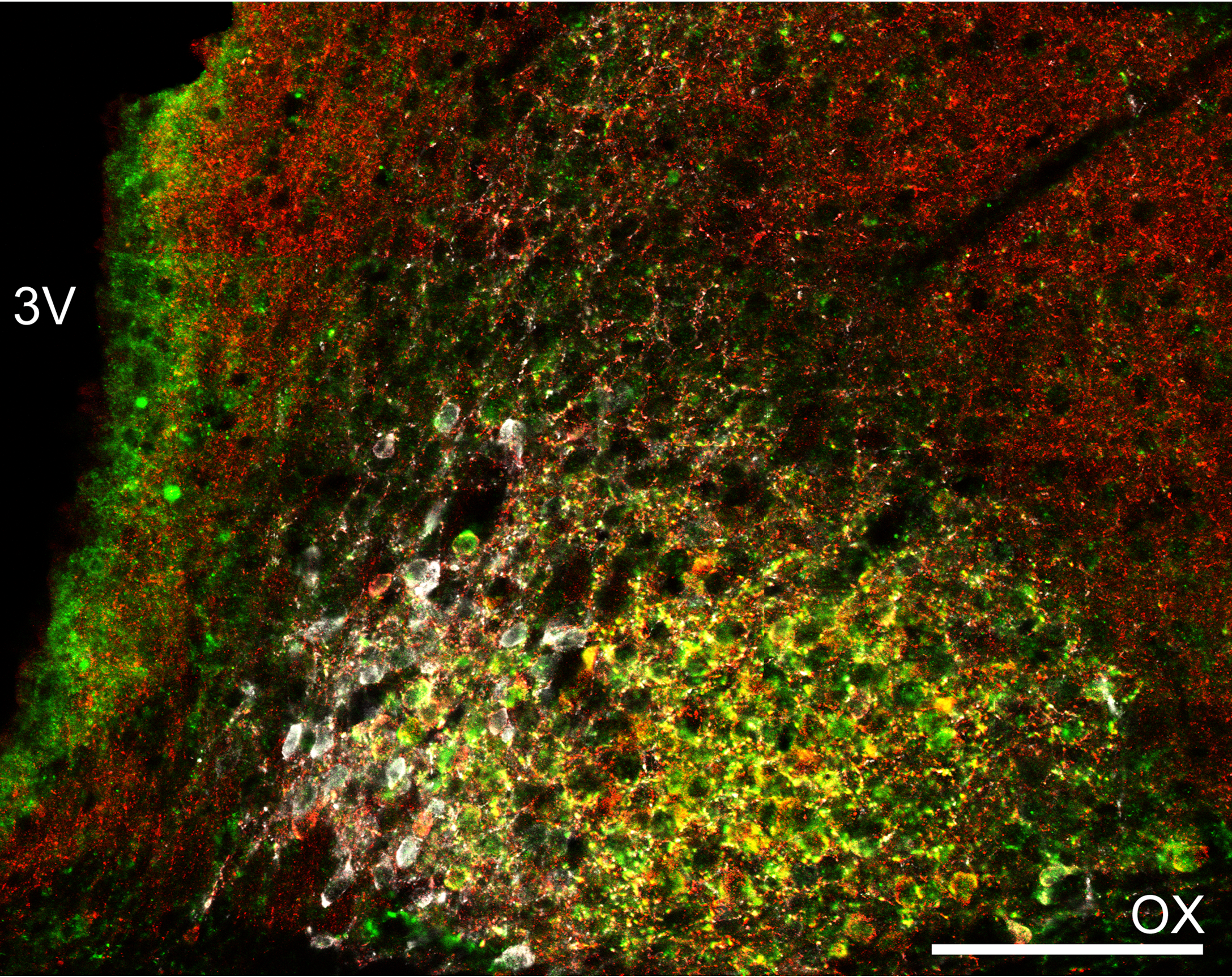

Supplement: Figure S3 — Enlarged confocal image of VIP/GRP/little SAAS immunostained rat rostro-medial SCN. Enlargement of Figure 2I reveals expression of respective peptides in this quadrant of the rat SCN: VIP (white), GRP (green), little SAAS (red), VIP/little SAAS (pink), and GRP/little SAAS (yellow). 3V, third ventricle; OX, optic chiasm. Single optical section, z = 0.11 µm. Scale bar = 100 µm. (9.99 MB TIF) [file pone.0012612.s003.tif]

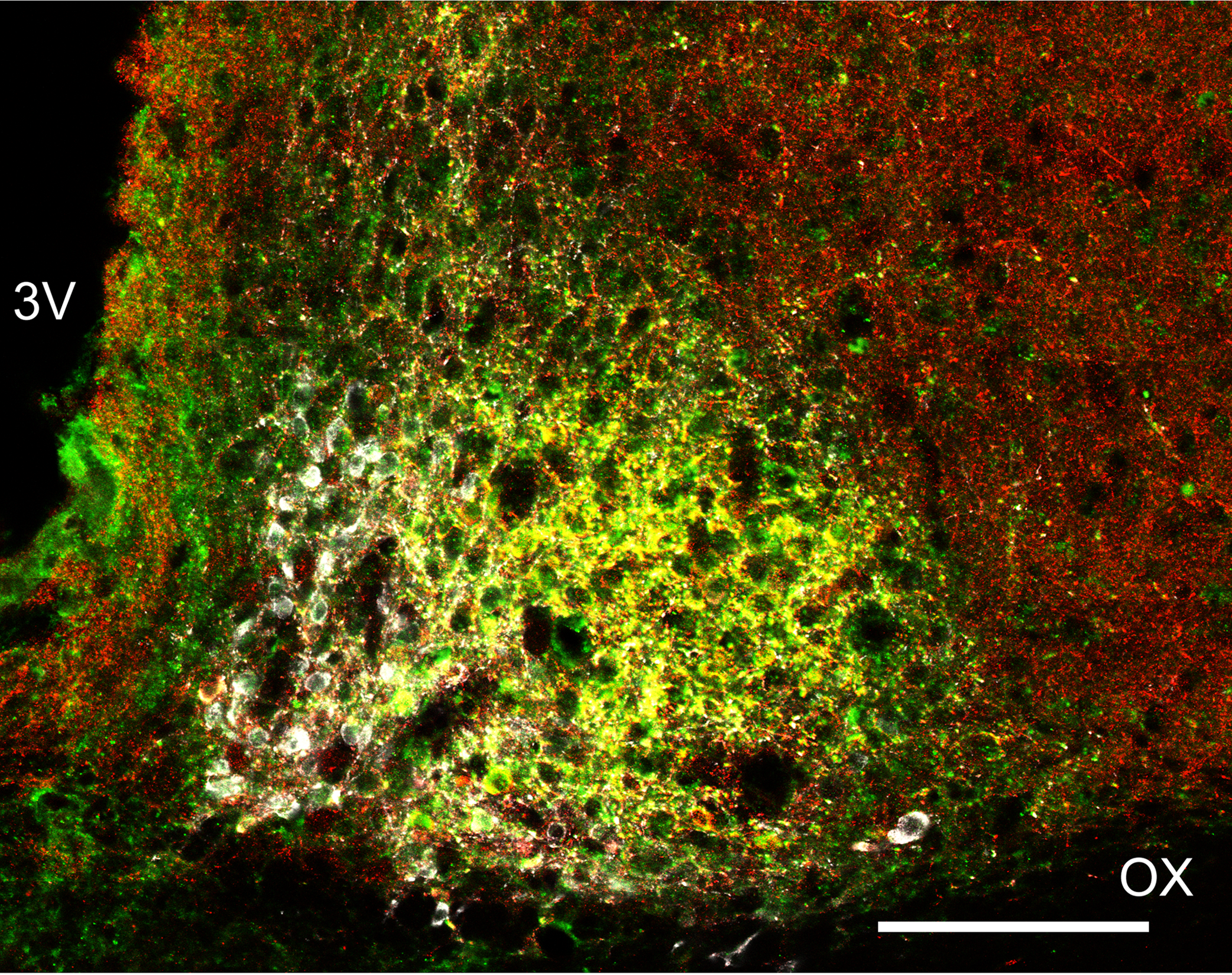

Supplement: Figure S4 — Enlarged confocal image of VIP/GRP/little SAAS immunostained rat medio-caudal SCN. Enlargement of Figure 2N reveals expression of respective peptides in this quadrant of the rat SCN: VIP (white), GRP (green), little SAAS (red), VIP/little SAAS (pink), and GRP/little SAAS (yellow). 3V, third ventricle; OX, optic chiasm. Single optical section, z = 0.11 µm. Scale bar = 100 µm. (9.39 MB TIF) [file pone.0012612.s004.tif]

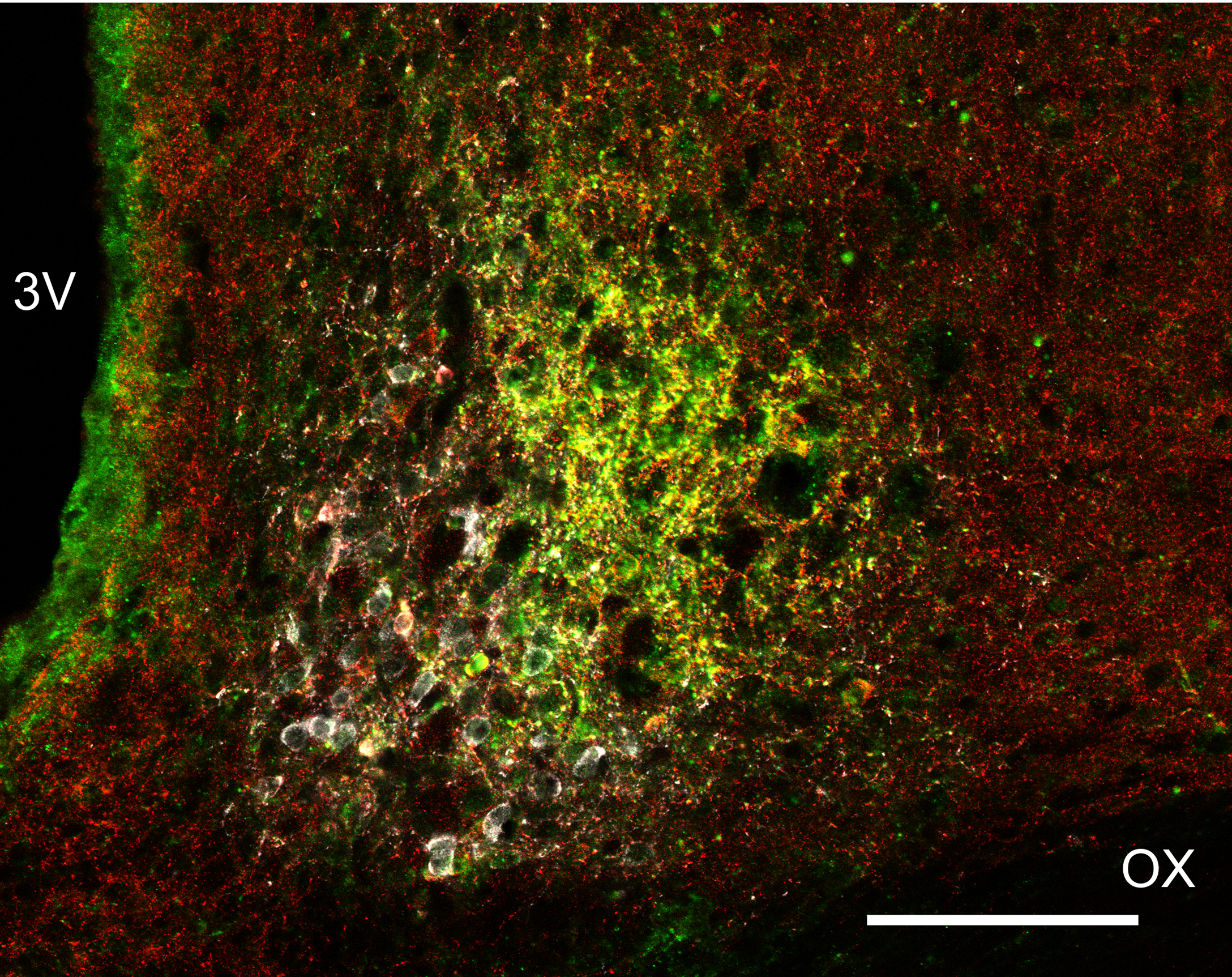

Supplement: Figure S5 — Enlarged confocal image of VIP/GRP/little SAAS immunostained rat caudal SCN. Enlargement of Figure 2S reveals expression of respective peptides in this quadrant of the rat SCN: VIP (white), GRP (green), little SAAS (red), VIP/little SAAS (pink), and GRP/little SAAS (yellow). 3V, third ventricle; OX, optic chiasm. Single optical section, z = 0.11 µm. Scale bar = 100 µm. (9.80 MB TIF) [file pone.0012612.s005.tif]
